# Prevalence of Vitamin-D deficiency is related to severity of liver damage in Hepatitis-C patients

**Published:** 2020

**Authors:** Ayesha Shahid

**Affiliations:** Dr. Ayesha Shahid Jinnah Sindh Medical University Karachi. Pakistan. Email: shahidayesha56@yahoo.com

This refers to the manuscript by Falak S, Aftab L, Saeed M, Islam A. entitled Prevalence of Vitamin-D deficiency is related to severity of liver damage in Hepatitis-C patients. Pak J Med Sci. 2020;36 (3):445-450

I would like to point out some short comings of the aforementioned study. The authors have not taken into account the geographical location of the city under study and the physiological variants such as skin color, sun exposure, clothing style, sun screen use and the nutritional status of the patient, all of which can influence the vitamin D measurement. The seasonality of the time of measurement of vitamin D, which has been found as an important pre analytical factor to achieve the diagnosis of vitamin D deficiency^1^-^3^ has also not been mentioned.^1^ Similarly, the impact of renal function on vitamin D absorption was not taken into account and thus no measurement of Glomerular filtration rate estimation (eGFR) was made^1^. The authors have not cared to mention their criterion for labeling the patient cirrhotic as per any scoring system, degree of fibrosis or through liver biopsy or any lab values, neither have they mentioned the phase of treatment of the patient.

While measuring vitamin D values, It seems logical to assess the levels of extra hepatic albumin for it is believed to be an independent risk factor for vitamin D inadequacy and deficiency.^1^ One unit increase in albumin has been predicted to cause a proportional increase of 25 nmol/l in the overall serum 25(OH)D concentration, therefore, vitamin D-binding protein (DBP) and albumin are important confounders of the total circulatory 25(OH)D. About 90 % of which is bound with DBP, 9·9 % is loosely bound with albumin, and only about 0·1 % stays in free-circulating fraction.^1^

Genotype of the HCV and the genetic influence on vitamin D metabolism cannot be overlooked as an integral factor which could affect the deficiency and also severity of fibrosis and response to treatment^2^, which the authors have also failed to discuss.
